# Est16, a New Esterase Isolated from a Metagenomic Library of a Microbial Consortium Specializing in Diesel Oil Degradation

**DOI:** 10.1371/journal.pone.0133723

**Published:** 2015-07-27

**Authors:** Mariana Rangel Pereira, Gustavo Fernando Mercaldi, Thaís Carvalho Maester, Andrea Balan, Eliana Gertrudes de Macedo Lemos

**Affiliations:** 1 National Laboratory of Biosciences (LNBio), Brazilian Center for Research in Energy and Materials (CNPEM), Campinas, São Paulo State, Brazil; 2 University of São Paulo, São Paulo, São Paulo State, Brazil; 3 Department of Technology, São Paulo State University, Jaboticabal, São Paulo State, Brazil; 4 Institute of Biology, University of Campinas, Campinas, São Paulo State, Brazil; 5 Department of Microbiology, Biomedical Sciences Institute II, University of São Paulo, São Paulo, São Paulo State, Brazil; Russian Academy of Sciences, Institute for Biological Instrumentation, RUSSIAN FEDERATION

## Abstract

Lipolytic enzymes have attracted attention from a global market because they show enormous biotechnological potential for applications such as detergent production, leather processing, cosmetics production, and use in perfumes and biodiesel. Due to the intense demand for biocatalysts, a metagenomic approach provides methods of identifying new enzymes. In this study, an esterase designated as Est16 was selected from 4224 clones of a fosmid metagenomic library, revealing an 87% amino acid identity with an esterase/lipase (accession number ADM63076.1) from an uncultured bacterium. Phylogenetic studies showed that the enzyme belongs to family V of bacterial lipolytic enzymes and has sequence and structural similarities with an aryl-esterase from *Pseudomonas fluorescens* and a patented Anti-Kazlauskas lipase (patent number US20050153404). The protein was expressed and purified as a highly soluble, thermally stable enzyme that showed a preference for basic pH. Est16 exhibited activity toward a wide range of substrates and the highest catalytic efficiency against *p*-nitrophenyl butyrate and *p*-nitrophenyl valerate. Est16 also showed tolerance to the presence of organic solvents, detergents and metals. Based on molecular modeling, we showed that the large alpha-beta domain is conserved in the patented enzymes but not the substrate pocket. Here, it was demonstrated that a metagenomic approach is suitable for discovering the lipolytic enzyme diversity and that Est16 has the biotechnological potential for use in industrial processes.

## Introduction

The global trade of industrial enzymes has been estimated to be 2.3 billion dollars [1]. Currently, the enzyme market has been estimated to be € 3.4 billion with an annual growth of 6.5 to 10% [2]. Within this market, lipolytic enzymes have attracted enormous attention due to their wide biotechnological applications. They are members of the broad family of proteins containing an alpha/beta hydrolase fold and can be classified according to their substrate preferences as lipases (EC 3.1.1.3) that hydrolyse water-insoluble long-chain acylglycerols (C > 10) or esterases (EC 3.1.1.1) that hydrolyse water-soluble short-chain acylglycerols (C ≤ 10).

Lipolytic enzymes are involved in catalyzing esters hydrolysis, ester synthesis, transesterification and other reactions. They can be used for the production of fine chemical syntheses [3], flavor compounds [4,5], antioxidants and perfumes [6]. Moreover, esterases can show high regio- and stereospecificity, can hydrolyse a wide range of substrates, do not require the presence of cofactors, and can be stable and active in organic solvents [7,8].

In view of the high demand for biocatalysts in biotechnology, new experimental approaches have been developed over the last few years to search for efficient molecules. Metagenomics is a cultivation-independent method that gives access to the collective genome [9] and to uncultured microorganisms, which is a vast source for gene exploration in biotechnology. In recent years, several genes encoding lipolytic enzymes were identified in metagenomic libraries from different environmental samples [10–16]. Additionally, lipolytic enzymes that display resistance to organic solvents [8,17] or salt tolerance [18] and show high potential for use in industrial processes could be prospected through metagenomic approaches.

Despite the interesting discoveries of new lipolytic enzymes from the metagenome, most enzymes remain uncharacterized. From the first screening for lipolytic enzymes in 2000 until 2009, 76 positive clones of new esterases or lipases were identified, but only 11 were overexpressed and purified for biochemical characterization [19].

Here, the cloning, overexpression, functional characterization and preliminary structural analysis of a novel esterase, Est16, is reported. Est16 was isolated from a metagenomic library of a microbial consortium specialized for diesel oil degradation. The results showed that Est16 is a new esterase member of Family V of bacterial lipolytic enzymes and shares some structural similarities with an aryl-esterase from *Pseudomonas fluorescens* [20] and a patented Anti-Kazlauskas lipase (patent number US20050153404). Est16 was expressed in *Escherichia coli* cells as a soluble, monodisperse and highly stable protein. Kinetics assays revealed that Est16 is active against a broad range of substrates with short and long acyl chains, but has the highest catalytic efficiency against *p*-nitrophenyl butyrate (C_4_) and *p*-nitrophenyl valerate (C_5_). In addition, the enzyme activity was maintained at different pHs and in the presence of detergents, organic solvents and ions. Altogether, these data show that Est16 presents a set of characteristics that make it an interesting candidate for biotechnological applications.

## Methods

### Sampling

As in a previous work, a soil sample was collected from a region contaminated with petroleum hydrocarbons from a factory of automotive lubricants in the city of Ribeirão Preto, São Paulo State, Brazil [21]. The soil was used to develop a microbial consortium at the Department of Biology at the University of São Paulo State, Jaboticabal Campus [21], which had the bacterial diversity analyzed [22]. From this microbial consortium, a metagenomic library of fosmid vectors was built in the Biochemistry Laboratory of Microorganisms and Plants (LBMP) in the Department of Technology at the same university. In this work, the 4224 clones obtained were used as source for prospecting lipolytic enzymes.

### Screening for lipolytic activity

The fosmid clones from the DNA metagenomic library were submitted to lipolytic activity screening on Luria Bertani (LB) agar Petri dishes containing 1% tributyrin, 1% gumarabic and 12.5 μg/mL chloramphenicol. Arabinose (0.001%) was added to induce a high copy number of the plasmids based on the oriV/trfA system [23]. As a negative control, *E*. *coli* EPI300 cells (Epicentre, Technologies, Madison, WI, USA) carrying the pCC2FOS vector supplied by a kit were used. The plates were incubated at 37°C for 3 days and then were transferred to 4°C for up to 7 days. The presence of a halo around the colonies was indicative of substrate hydrolysis and lipolytic activity by the clones, which were then replicated to confirm the result.

### Selection of the clone and sub-library construction

A fosmid clone that formed a clear halo was selected for construction of the sub-library and to identify the proper gene encoding the putative esterase/lipase. The DNA clone was fragmented using the nebulization method [24] and the material was applied to a 1% agarose gel for electrophoresis. The 1 to 3 kb fragments of interest were recovered, purified from the gel with the Geneclean III kit (BIO 101 Inc., Vista, CA, USA), and cloned into the pUC19 vector (Fermentas, Burlington, ON, Canada), which was previously digested with *Sma*I (Fermentas) and dephosphorylated with Shrimp Alkaline Phosphatase (New England Biolabs, Ipswich, MA, USA). The ligation mixture was used for transformation of *E*. *coli* DH5α cells and the selection of the clones was performed as previously described using 96-wells plates supplemented with LB media and 70 μg/mL ampicillin [24].

### DNA sequencing, gene annotation and qualitative analysis of the Est16 activity

The plasmid DNA extracted from the subclones of the sub-library was sequenced in both directions using the M13-Forward and M13-Reverse primers. For sequencing the fosmid ends, the pCC2F and pCC2R primers (Epicentre) were used. Sequencing was performed on an ABI 3100 instrument (Applied Biosystems, Foster City, CA, USA) and the electropherograms were analyzed with the aid of the PhredPhrap program [25, 26]. The contig was compared with the sequences deposited in the National Center for Biotechnology Information (NCBI) using the local alignment BLAST tool [27] against the nucleotide collection [(nr/nt), December 2014]. Gene annotation was performed using the ORF Finder from NCBI (http://www.ncbi.nlm.nih.gov/projects/gorf/) and the ORF function prediction was performed using a BLASTX query against the non-redundant bank protein (nr), taking into account the maximum score, the query coverage, the E-value (1e^-15^) and the presence of conserved domains. To check if the subclone containing the putative esterase/lipase gene presented lipolytic activity, bacterial colonies were plated onto Petri dishes containing LB-agar with 1% tributyrin, 1% gumarabic and 70 μg/mL ampicillin, and incubated at 37°C for 3 days and then 4°C for 4 days. As mentioned before, the activity was evidenced by the appearance of a clear halo around the colonies on the plates.

### Phylogenetic analysis

Two distinct phylogenetic trees were built to analyze the phylogenetic relationship of Est16. The first one was built with the 34 previously described lipolytic enzymes from the eight families proposed by Arpigny and Jaeger [28] (S1 Table), which were obtained from NCBI, and the second one with 18 sequences from patented enzymes that show industrial applications and with a sequence of an aryl-esterase from *Pseudomonas fluorescens* (PDB code 1VA4) [20]. The sequences were aligned with Est16 using the ClustalW program [29] (pairwise alignment with gap opening 35 and extension 0.75) and the alignment file was submitted to Mr. Bayes 3.2 [30] and MEGA6 [31] to estimulate a suitable evolutinoray model in the construction of the filogenetic tree. The method of phylogenetic construction used was the Maximum Likelihood with a bootstrap of 1,000 replicates and the WAG matrix of amino acid substitution.

The choice of patented sequences was based on the literature data [32] and industrial applications using the Orbit database at the Questel website (https://www.questel.com). The patents were selected on Orbit database combining keywords in the input plataform related to lipase or esterase and some biotechnological application in which they are used. After the search, the sequences were obtained from the GenomeQuest website (http://www.genomequest.com/) (S2 Table) and aligned for tree preparation, which was built as mentioned before.

### Molecular modeling of Est16 and structural analysis

The molecular model of Est16 was built with the Modeller 9v4 program [33] using the structural coordinates of an aryl-esterase from *Pseudomonas fluorescens* (PDB code 1VA4) [20] as a model. In addition, to evaluate the structural features of Est16 in comparison with the patented Anti-Kazlauskas lipase (patent number US20050153404), a model of this structure was built using the structural coordinates of an esterase from *Pseudomonas fluorescens* (PDB code 3IA2) [34] that shares 22.8% sequence identity with the patented enzyme. Then, the superposition of the structures was performed using COOT [35]. The sequence comparison and the structural analysis were performed using ClustalW [29] and the Pymol program [36], respectively. All final figures were prepared with Pymol.

### Cloning, expression and purification of Est16

The *est16* gene was amplified by Polymerase Chain Reaction (PCR) with the Pfu DNA Polymerase enzyme (Fermentas) using the following primers: Forward 5’ CTGAATTCTCCATGCCGCAGGTTCAG 3’ and Reverse 5’ GGGCTCGAGCGTCACTCCGCC 3’, which had sites for the restriction enzymes *Eco*RI and *Xho*I, respectively (underlined). The amplified fragment consisting of 917 bp was previously digested with the mentioned enzymes and cloned into the pET28a vector (Novagen, Gibbstown, NJ, USA) for protein expression. The recombinant pET28a-*est16* plasmid was used for transformation of chemically competent *E*. *coli* BL21 (DE3) cells. For expression tests, a pre-inoculum of the cells was grown at 37°C for 20 hours in 5 mL of LB media containing 50 μg/mL kanamycin. This pre-inoculum was then used to inoculate 500 mL of LB (50 μg/mL kanamycin). The culture was grown under shaking at 250 r.p.m. at 37°C until the OD_600nm_ reached 0.5–0.6, at which point 0.1 mM isopropyl-β-D thiogalactopyranoside (IPTG) was added to induce protein expression at 28°C for 20 hours. After this period, the cells were centrifuged at 8000 g for 10 minutes at 4°C and resuspended in 35 mL of lysis buffer [10 mM Tris-HCl pH 8.0, 500 mM NaCl, 10 mM imidazole, 10 mM β-mercaptoethanol, 10% (v/v) glycerol and 0.25% (w/v) nonidet P-40]. The material was maintained in an ice bath for 1 hour with 1 mM DL-Dithiothreitol (DTT) and 4 μg/mL of lysozyme. Ultrasonication was performed for 10 cycles of 10 pulses at a 40% amplitude with 10 second intervals using an LB-750 sonicator (Labometric, Leiria, Portugal). The obtained extract was centrifuged at 17,000 g for 45 minutes at 4°C to pellet the cell debris. The expression and solubility of the protein was confirmed after loading the non-induced, induced, extract and pellet samples into an SDS-PAGE gel. After the solubility of Est16 was verified, 3 mL of Ni-NTA resin (Qiagen, Hilden, Germany), which was previously equilibrated with the purification buffer [50 mM Tris-HCl pH 8.0, 500 mM NaCl, 10 mM imidazole and 10% (v/v) glycerol], was added to the soluble extracts for protein purification using immobilized metal affinity chromatography (IMAC). The sample was incubated for 1 hour at 4°C under agitation before it was loaded into a gravity flow column. After the wash of the resin with 10 column volumes of purification buffer containing 10 mM of imidazole, the protein was eluted in a stepwise imidazole gradient (50–500 mM imidazole). The eluted fractions were concentrated with Amicon Ultra-15 filters (Merck Millipore, Billeria, MA, USA) under centrifugation at 3000 g at 4°C. To eliminate the contaminants, additional size-exclusion chromatography (SEC) was performed using the Hiload 16/60 Superdex 200 column (GE Healthcare Bio-Sciences, Uppsala, Sweden). This purification was performed with the buffer (20 mM Tris-HCl pH 8.0; 50 mM NaCl; 5% glycerol) at 20°C. All samples were analyzed using SDS-PAGE and the protein concentration was measured using a Nanodrop ND-1000 spectro-photometer (Nanodrop Technologies, Wilmington, DE, USA).

### Tributyrin assay in Petri dishes

The pure protein obtained from size-exclusion chromatography was tested in different pHs (7.0, 8.0 and 9.0) against tributyrin in Petri dishes containing 0.23% (w/v) Tris; 1.2% (w/v) agar; and 1% (v/v) tributyrin. To each dish was added 1 μg of Est16 protein and they were incubated at 37°C for 1 day in order to verify the substrate hidrolysis in these pHs.

### Measuring the enzymatic characteristics of Est16

The Est16 activity was determined via hydrolysis of *p*-nitrophenol (*p-*NP) ester substrates by measuring the formation of *p*-nitrophenol. The amount of *p*-nitrophenol released was quantified by absorbance at 405 nm (ε_pNP_ = 17,000 M^-1^.cm^-1^) using a 2104 Envision Multilabel Reader (PerkinElmer, Waltham, Massachusetts, USA). All assays were carried out in triplicate in 96-well plates with a final volume of 100 μL. Pipetting steps were performed using a Janus Varispan automated liquid handler (PerkinElmer). Unless otherwise indicated, the reactions were carried out at 30°C using 15 nM of the enzyme and 1 mM of substrate in 50 mM Tris-HCl pH 8.0 and 0.3% (v/v) triton X-100. For the analysis of the substrate specificity range of the purified Est16, the hydrolysis of the following *p*-NP-fatty acyl esters (Sigma, MI, USA) was measured: butyrate (C_4_), valerate (C_5_), octanoate (C_8_), decanoate (C_10_), dodecanoate (C_12_), myristate (C_14_) and palmitate (C_16_). The optimum temperature for the enzyme activity was evaluated using *p*-NP valerate and *p*-NP octanoate by measuring the production of *p*-nitrophenol in the range of 25 to 65°C (5°C steps) for 10 minutes using an EnSpire Multimode Plate Reader (PerkinElmer). The effect of the pH on the activity of Est16 against *p*-NP butyrate was measured at pH 5.0 to 11.0 using the following buffers at 50 mM: sodium citrate pH 5.0 and 6.0; Tris-HCl pH 7.0, 8.0 and 9.0; N-cyclohexyl-2-aminoethanesulfonic (CHES) pH 10.0; and sodium phosphate pH 11.0. All reactions contained 0.3% (v/v) triton X-100.

The influence of metal ions on the performance of Est16 was evaluated using the substrate *p*-NP butyrate, and the activity was measured in the presence of 0.5 mM of the following reagents: Ca^2+^, Co^2+^, K^+^, Mg^2+^, Mn^2+^, Ni^2+^, and Zn^2+^. An assay with 0.5 mM of ethylenediaminetetraacetic acid (EDTA) was also performed. Controls of the reactions were developed for all conditions and references were obtained in the absence of metals, ions or EDTA. Lastly, to evaluate the tolerance of the enzyme to detergents and solvents, the Est16 hydrolysis of *p*-NP butyrate was evaluated in the presence of the detergents tween 20 and triton X-100 [0.3125 to 5% (v/v)] and with the solvents dimethylformamide (DMF) and dimethyl sulfoxide (DMSO) [2.5 to 10% (v/v)]. Neither the solvents nor the detergents were added to the control samples. The reactions were carried out in 50 mM Tris-HCl pH 8.0.

The kinetic parameters of Est16 were calculated from different reactions. The initial rate of the reaction was measured using 15 nM of the enzyme for substrates C_4–_C_10_ and 50 nM for substrates C_12–_C_16_. Two set of substrate concentrations were used depending on the chain length: 0.0078 mM to 1 mM for *p*-NP acetate, butyrate, valerate, and octanoate, and 0.0156 mM to 2 mM for *p*-NP decanoate, dodecanoate, myristate and palmitate. The kinetic parameters for each substrate were obtained by non-linear regression of the data using the following equation: *V* = V_max_ * [S]/(K_*m*_ + [S]), where *V* is the rate, V_max_ is the maximum enzyme rate, S is the substrate concentration, and K_*m*_ is the Michaelis-Menten constant.

All data obtained from the characterization of Est16 were analyzed using the R software. ANOVA and Tukey’s test at 5% probablility were used to compare the treatment methods.

### Circular dichroism

Circular dichroism (CD) measurements were carried out with a JASCO J-810 spectropolarimeter equipped with a Peltier-type temperature controller and a thermostatic cell holder, which was interfaced with a thermostatic bath. To verify the influence of pH, the Est16 protein was diluted in different buffers (5 mM sodium phosphate pH 7.0; 5 mM sodium phosphate pH 8.0; 5 mM CHES pH 9.0) and submitted to the CD analysis. Thirty consecutive scans from 195 to 260 nm were compiled and the average spectra were stored. The data were corrected for the baseline contribution of the buffer. Thermal unfolding experiments were performed in 1-mm-long cells by increasing the temperature from 20°C to 110°C. The temperature was allowed to equilibrate before each spectrum was recorded. The T*m* represents the temperature at the midpoint of the unfolding transition. CD of Est16 was performed at the concentration of 5.64 μmol of protein.

### Nucleotide sequence accession number

The DNA sequence of the *est16* gene was deposited in GenBank under the accession number KM882609‏.

## Results and Discussion

### Metagenomic library construction and screening for lipolytic activity

**Fig 1:**
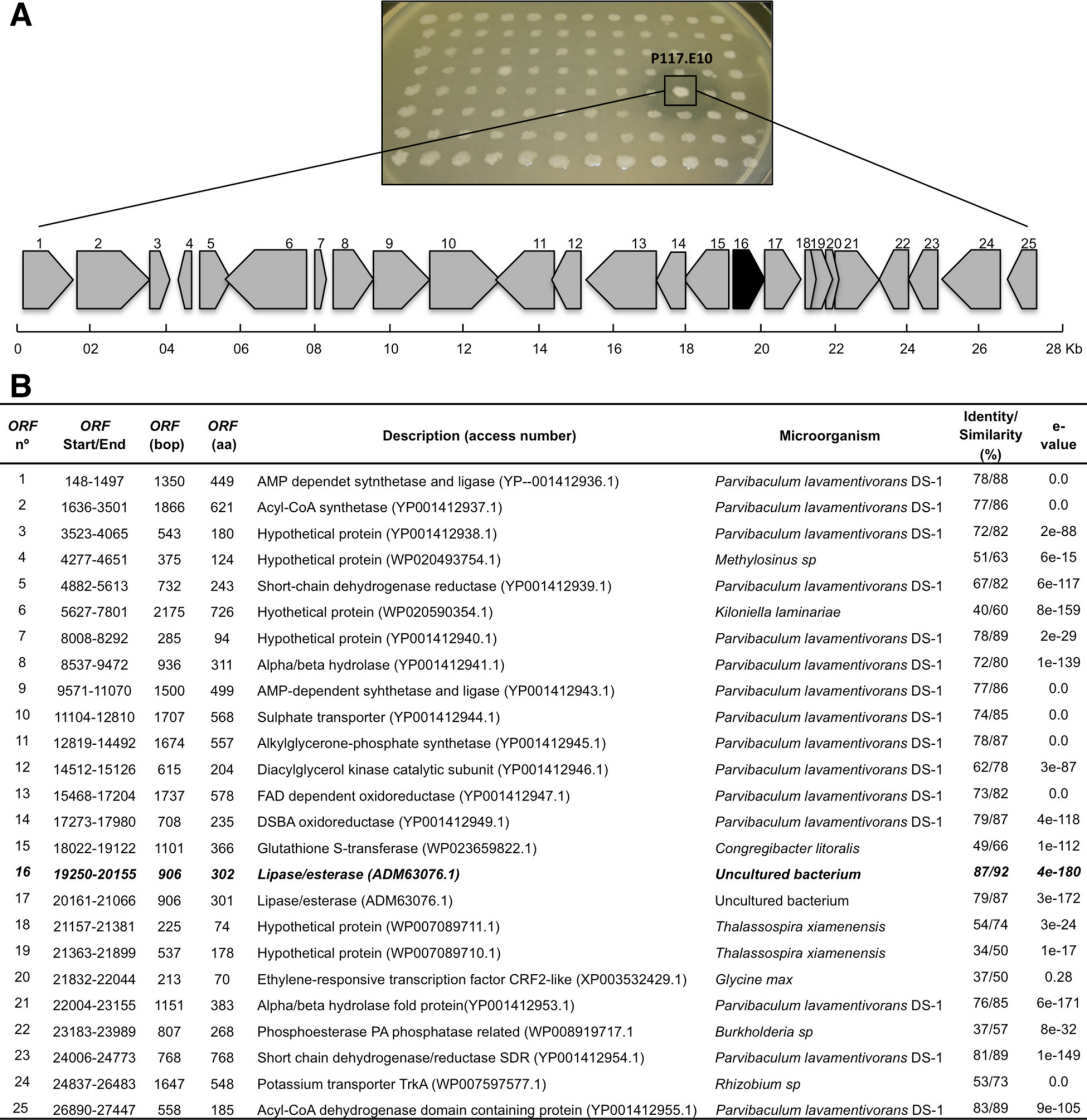
Lipolytic activity and gene annotation of the Pl17.E10 clone. **(A)** Clones obtained from the metagenomic approach were cultivated in a Petri dish containing LB media supplemented with 1% (v/v) tributyrin, 1% (w/v) gumarabic, 0.00125% (v/v) chloramphenicol and 0.001% (v/v) arabinose. The plate was maintained at 37°C for three days and for four more days at 4°C. A clear halo was observed around the P117.E10 fosmid clone, evidencing its ability to degrade tributyrin. A detailed physical map of Pl17.E10 is shown, which consists of 25 identified ORFs depicted as arrows according to their location and direction in the fosmid vector. The black arrow indicates ORF16, which encodes the Est16 protein. **(B)** The characteristics of each ORF were obtained after using ORF Finder and the BLASTP search. The putative function of each ORF and their GenBank accession numbers are also shown.

A fosmid metagenomic library was constructed using high molecular weight DNA isolated from a microbial consortium specialized for the degradation of diesel oil. A total of 4224 clones were obtained with an average insert DNA size of 30 to 40 kb, representing a total size of 148 Mb of the total metagenomic DNA. All clones were screened for lipolytic activity based on the hydrolytic activity of emulsified tributyrin (1%), resulting in 30 positive lipolytic clones that showed a clear zone around the colonies, which corresponded to approximately one lipolytic gene per 4.9 Mb DNA (one positive clone per 140 tested clones). From one of the screening plates with 96 colonies, the Pl17.E10 clone exhibited strong lipolytic activity as evidenced by the clear halo (Fig 1A). The Pl17.E10 clone was chosen for construction of the sub-library and gene annotation. The fosmidial DNA of the P117.E10 clone was extracted and an insert of approximately 35 kb was subcloned. 672 subclones with 1 to 3 kb inserts were collected and the plasmid DNA was extracted and quantified for sequencing. The final contig was 27.485 bp and shared a 78% nucleotide sequence identity and 52% query coverage with *Parvibaculum lavamentivorans* (accession number CP000774.1). From 147 ORFs identified by the ORF Finder, 25 were selected using BLASTP (Fig 1B). From these, ORF16, which consists of 906 bp (302 amino acids, in bold), was identified as a putative gene encoding an esterase/lipase that was similar to an esterase/lipase from an uncultured bacterium (accession number ADM63076.1; 87% sequence identity) and a protein from *Parvibaculum lavamentivorans* DS-1 (accession number WP012110584.1; 82% sequence identity) with alpha/beta hydrolase fold. The ORF16 was chosen for further analysis and the corresponding protein was designated Est16.

### Est16 is most close to members of families V and structuraly similar to the patented Anti-Kazlauskas lipase

To first evaluate the phylogenetic relationship between Est16 and the 34 sequences from lipolytic enzymes representing the families proposed by Arpigny and Jaeger [28], a phylogenetic tree was built using the MEGA6 program after the alignment of the Est16 amino acid sequence with all sequences (S1 Fig). Est16 is related to a lipolytic enzyme from *Sulfolobus acidocaldarius* (accession number AAC67392.1), a lipase from *Psychrobacter immobilis* (accession number CAA47949.1) and a lipase from *Moraxella* sp. (accession number CAA37863.1), sharing 23%, 18% and 19% amino acid sequence identity with Est16, respectively (S1A Fig). According to Arpigny and Jaeger [28], these enzymes belong to family V of bacterial lipolytic enzymes. Est16 multiple sequence alignment with *Sulfolobus acidocaldarius* (accession number AAC67392.1) confirmed the conservation of the consensus region of family V and the residues around the catalytic triad formed by Ser118, Asp245 and His273. The catalytic nucleophile Ser is located in the central portion of the conserved motif G-X-S-M-G-G (S1B Fig), which is a sequence pattern of family V [28]. Interestingly, another enzyme that belongs to family V, an esterase/lipase from *Haemophilus influenzae* (accession number AAC21862.1) showed a close relationship with lipases from *Bacillus pumilus* (accession number CAA02196.1) and *Bacillus subtilis* (accession number AAA22574.1), which are members of family I. A similar result has already been observed before [16], where members of family I and family V were closely located, suggesting that these groups probably diverged from a common ancestor.

**Fig 2:**
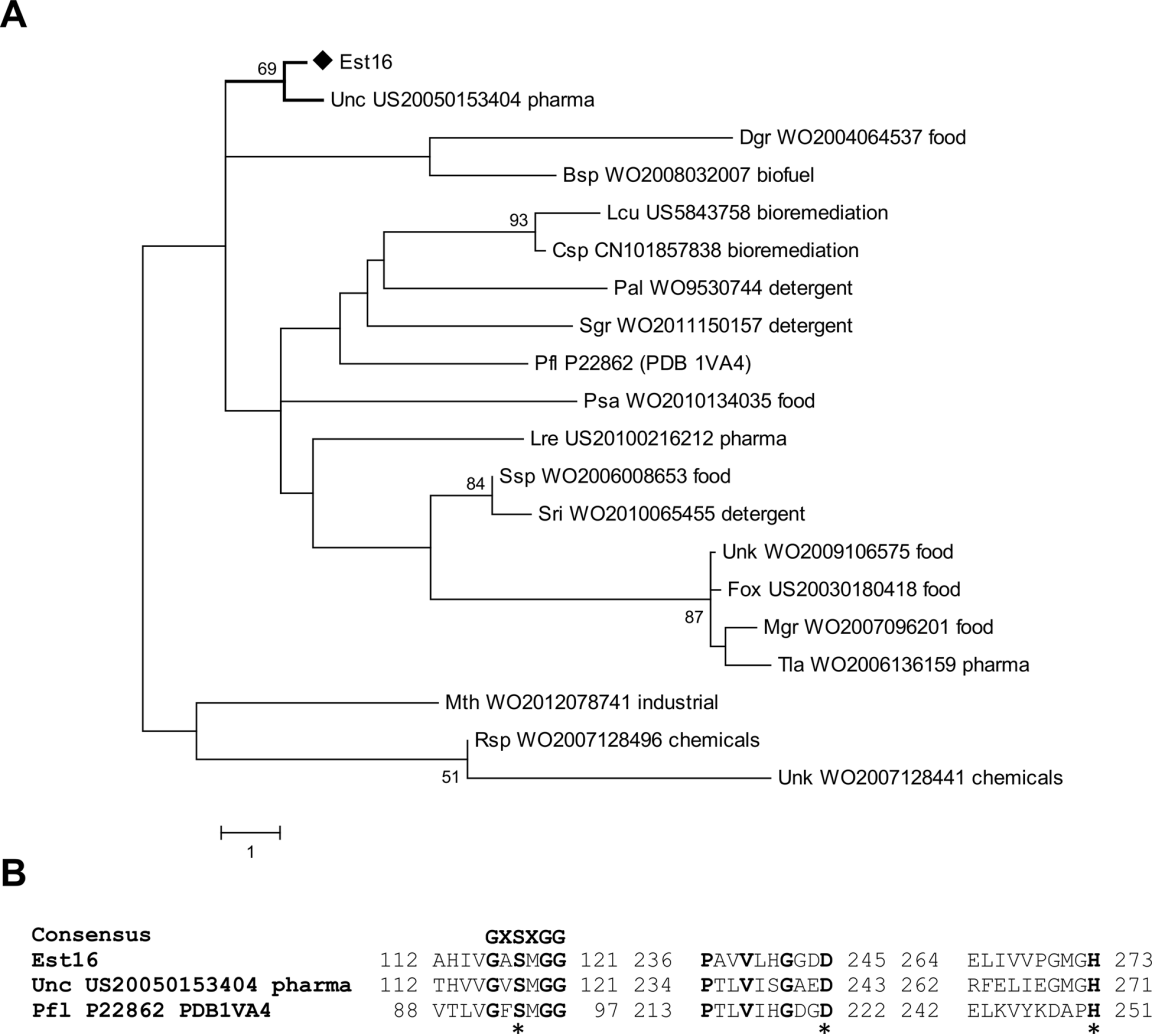
Phylogenetic tree and comparison of Est16 with esterases/lipases showing their biotechnological applications. **(A)** A phylogenetic tree of 18 sequences from patented esterases/lipases, the sequence of aryl-esterase (PDB code 1VA4) and Est16 was generated using the Maximum Likehood method (MEGA6.0) with a bootstrap of 1,000 replicates. The estimated value of the shape parameter for the discrete Gamma Distribution is 16.1381. Substitution pattern and rates were estimated under the WAG model (+G) with 5 categories. **(B)** The amino acid sequence alignment of Est16 with Anti-Kazlauskas lipase (patent number US20050153404) and aryl-esterase (PDB code 1VA4). The residues involved in the substrate-pocket and family classification are shown in bold and the catalytic triad residues are denoted with asterisks (*). The consensus motif of family V is shown.

To compare Est16 with proteins with solved three-dimensional structures, we submitted the amino acid sequence of Est16 to BLASTP against the Protein Data Bank [PDB (http://www.rcsb.org/pdb/home/home.do)]. The results revealed that Est16 has structure similarity to an aryl-esterase from *Pseudomonas fluorescens* (PDB code 1VA4) [20] that shares a 22.7% amino acid sequence identity with Est16. To verify whether Est16 presented similarities with patented enzymes, we also built a second phylogenetic tree that included 18 sequences of esterases/lipases extracted from GenomeQuest and the sequence of the *P*. *fluorescens* aryl-esterase [20]. Interestingly, this second analysis corroborated the results of the BLASTP query against the PDB, revealing that Est16 is most closely related to three enzymes: an enzyme from *P*. *fluorescens*, the Anti-Kazlauskas lipase (patent number US20050153404) that is used to prepare enantiomer-enriched esters and enantioselectivities in a racemization catalyst; a lipase from *Digitalis grandiflora* (patent number WO2004064537) that is applied as an emulsifier in a foodstuff; and a lipase from *Bacillus* sp. (patent number WO2008032007) that is used in a method for biodiesel production (Fig 2). The tree revealed that, in general, the enzymes were grouped according to their applications.

### The structural features of Est16 model corroborates the sequence similarities with *Pseudomonas fluorescens* and the Anti-Kazlauskas lipase

**Fig 3:**
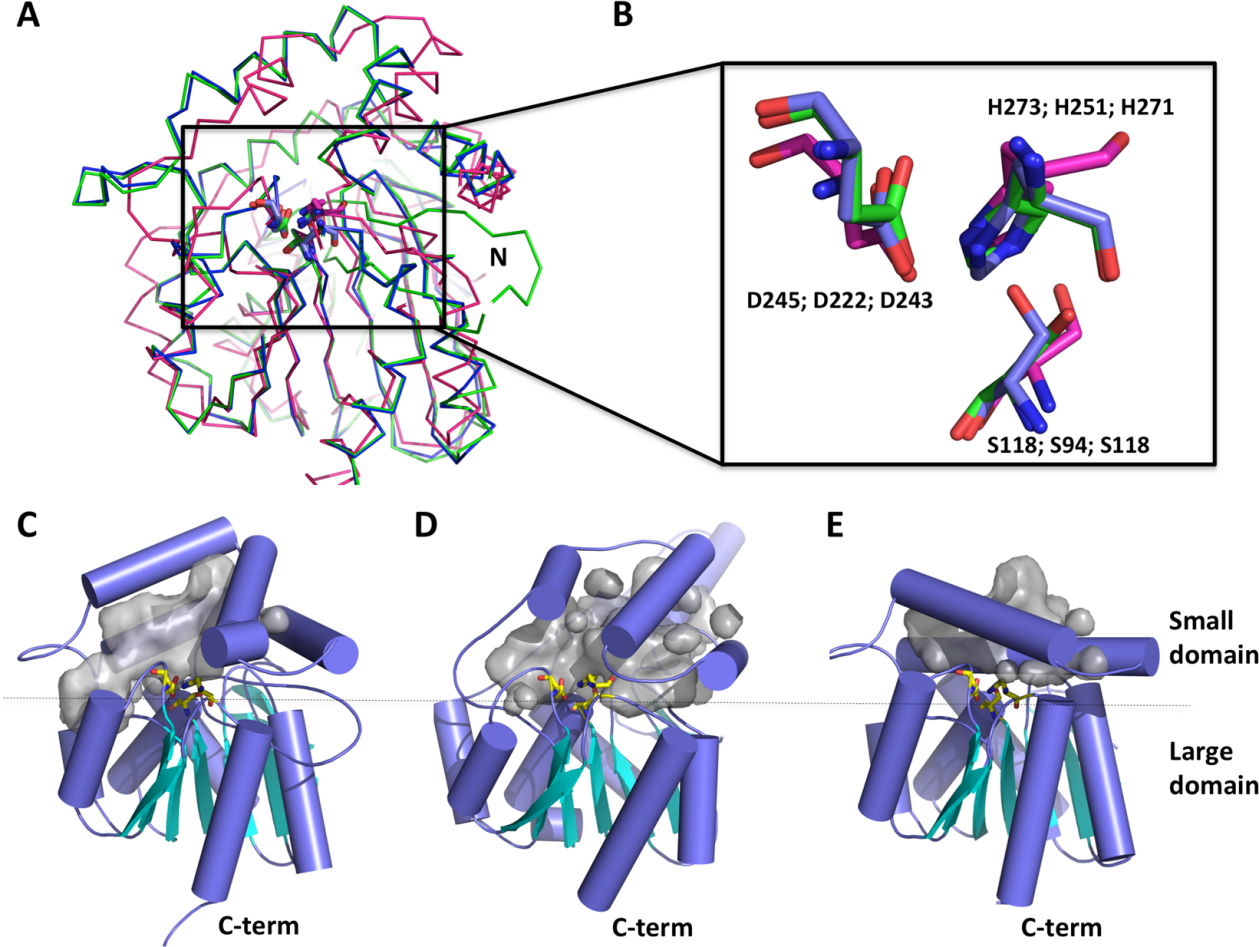
Structural features of the Est16 model in comparison with an esterase from *P*. *fluorescens* and the Anti-Kazlauskas lipase. **(A)** Ribbon representation of the Est16 model (green) superposed on the structures of *P*. *fluorescens* esterase (blue) and the patented lipase (patent number US20050153404) (purple) revealing the conservation of the alpha/beta fold. N is the N-terminal. **(B)** A detailed picture of the residues from the catalytic triad of the three enzymes showing the structural superposition. Residues are shown in the following order: Est16, *Pfl* (PDB code 1VA4) and Anti-Kazlauskas lipase. A comparative analysis of the domains and pockets of Est16 **(C)**, the Anti-Kazlauskas lipase model **(D)** and the structure of the *P*. *fluorescens* aryl-esterase **(E)** are shown in the cartoon. Helices are blue, β-sheets are cyan and the residues in the active site are shown as yellow sticks. The large and small domains are delimited in the three proteins.

As previously shown, the BLASTP query of the Est16 amino acid sequence versus the PDB confirmed the phylogenetic analysis and revealed the best score for the three-dimensional structure of the *Pseudomonas fluorescens* aryl-esterase (PDB code 1VA4) [20]. Using the structural coordinates of this enzyme, we built a three-dimensional model of Est16. Moreover, based on the close relationship between Est16 and the Anti-Kazlauskas lipase, we also modeled the structure of this patented enzyme for further structural analyses. Est16 model was nicely superposed onto the structures of the *P*. *fluorescens* and Anti-Kazlauskas lipases structures, revealing r.m.s.d. values of 2.8 Å and 3.6 Å, respectively. The three structures conserved the large alpha/beta domain that characterizes a hydrolase fold and sustains the smaller alpha-helical domain, which showed significant differences between the three structures (Fig 3A). The active sites of each enzyme are located between the two domains and the superposition revealed that the residues from the catalytic triad are located exactly in the same positions in the three structures irrespective of the structural differences (Fig 3B). For comparison of the full structures, we positioned the C-termini facing down maintaining the β-sheets in the horizontal plane, and depicted the catalytic triad as a yellow stick in between both domains. In this way, the differences in the substrate-binding pocket (in gray surface) are evidenced (Fig 3C–3E) due to the number and positioning of the α-helices. The arrangement and folding of these helices altered the size and dimensions of the substrate-binding pockets (Fig 3C–3E, gray surface). Compared to the Est16 substrate-binding pocket, the helices of the Anti-Kazlauskas lipase, which were almost parallel in orientation to those of Est16, formed a bigger channel compared to the small channel presented by the *P*. *fluorescens* enzyme. Due to these characteristics, this region can be considered important for substrate selectivity, enzymatic activity, or protein engineering using rational design or directed evolution targeting the enantioselectivity of the enzyme.

### Est16 was expressed and purified as a soluble, monodisperse and stable protein

**Fig 4:**
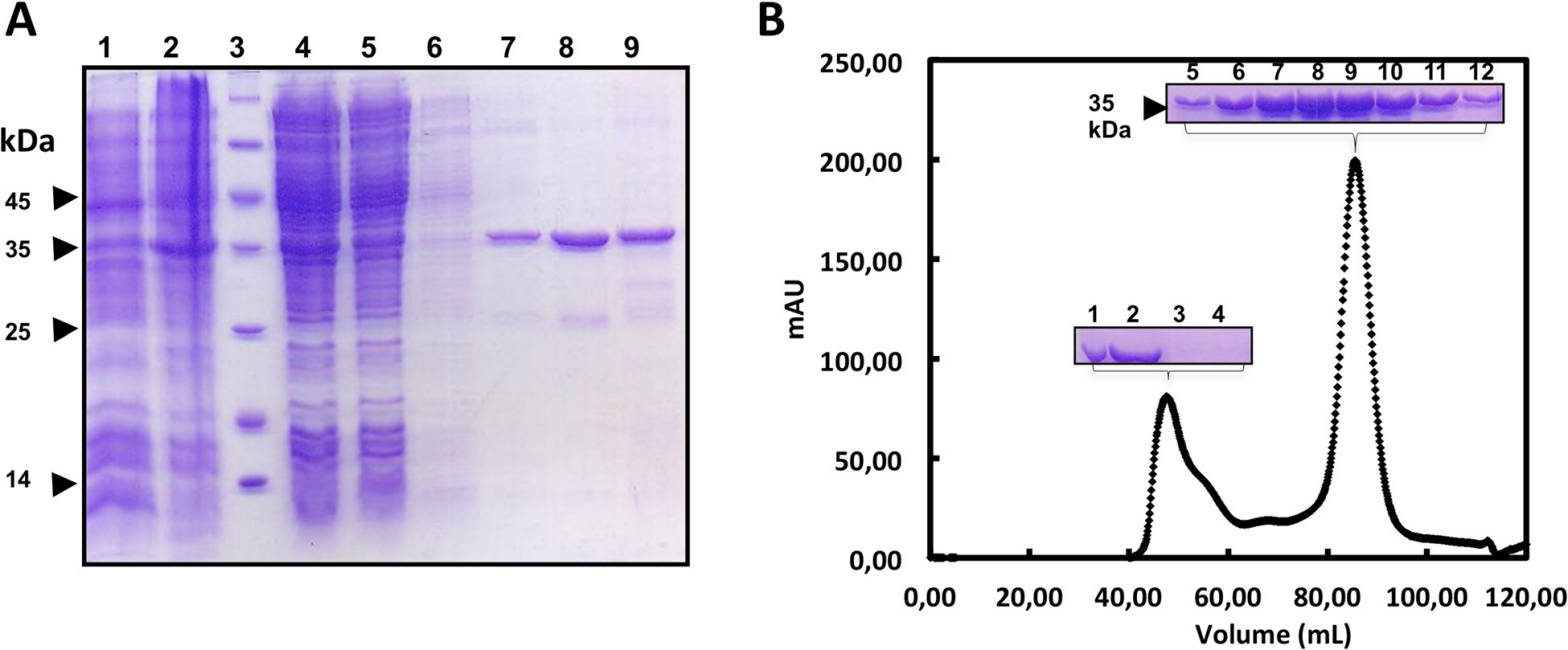
Production steps of Est16 as a soluble and stable protein. **(A)** 12% SDS-PAGE of the expression fractions and IMAC samples of Est16 from *E*. *coli* BL21 (DE3) cells carrying the pET28a-*est16* vector, which contained the *est16* gene. *E*. *coli* cells were grown in LB up to O.D._600nm_ = 0.5 at 37°C and then induced with 0.1 mM IPTG at 28°C for 20 hours. Lane 1: non-induced cells; Lane 2: induced cells; Lane 3: molecular weight marker (the kDa values are indicated in the picture); Lane 4: soluble extracts; Lane 5: flow-through fraction from the IMAC purification; Lane 6: 10 mM imidazole wash fraction; Lanes 7, 8 and 9: 50 mM, 100 mM and 1 M imidazole elution fractions, respectively. **(B)** The chromatogram obtained after the size-exclusion chromatography using a HiLoad 16/60 Superdex 200 column (GE Healthcare Bio-Sciences). The insets show the samples related to the peaks loaded into the 12% SDS-PAGE polyacrylamide gels. Lane 1, molecular weight standards; Lane 2: sample before SEC; Lanes 3–4: fractions from the first peak; Lanes 5–12: fractions from the second peak, which were concentrated for further spectroscopic and biochemical analyses.

The recombinant Est16 protein was expressed in the BL21 (DE3) strain of *E*. *coli* as a soluble protein genetically fused at the N-terminal end to a His_6_-Tag and the thrombin cleavage site encoded by the pET28a expression vector. The molecular mass of the recombinant Est16 protein was 35.4 kDa, as expected from its amino acid sequence. The maximum soluble protein yields, representing up to 60% of the total expressed protein, were achieved after 20 hours of cultivation in LB media at 28°C with aeration. The recombinant protein was first purified by immobilized metal affinity chromatography and eluted using a buffer containing 50 mM imidazole (Fig 4A), followed by one step of size exclusion chromatography (Fig 4B). Two peaks were obtained in the second purification step, but the DLS analysis showed that the protein was monodisperse only in the second, most abundant peak. The recombinant Est16 recovered at the end of the purification was concentrated and remained soluble and stable at high concentrations (more than 10 mg/mL) even after prolonged storage at 4°C. Its stability allowed for spectroscopic assays and further experiments to determine its kinetic parameters.

### Purified Est16 is active in basic pH and thermostable

**Fig 5:**
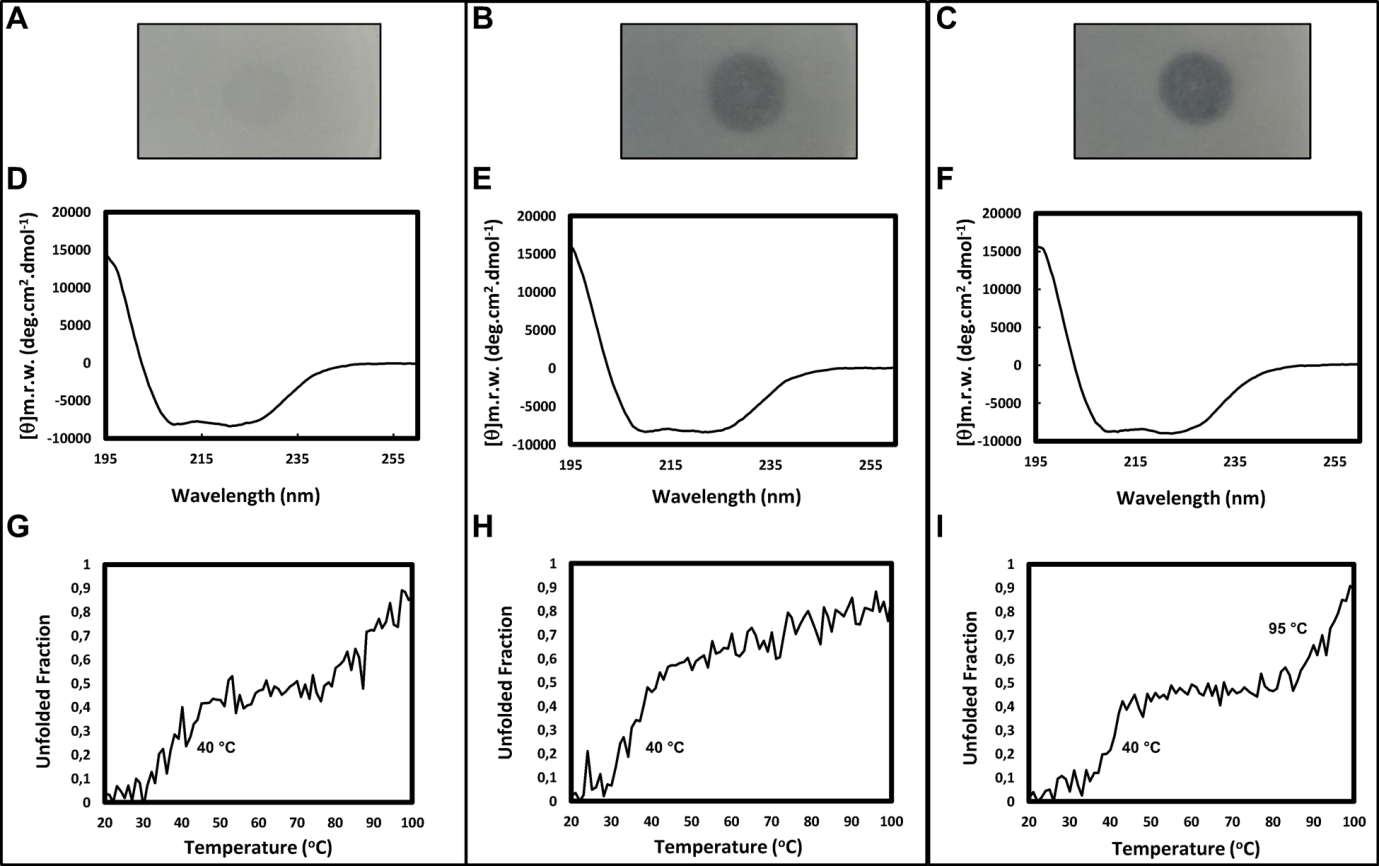
Tributyrin assay in Petri dishes and spectroscopic analyses of Est16. Tributyrin assays of Est16 (50 μg/mL) in Petri dishes containing 0.23% Tris-HCl (w/v), 1.2% agar (w/v) and 1% tributyrin at pH 7.0 **(A)**, pH 8.0 **(B)** and pH 9.0 **(C)**. Circular dichroism assays of Est16 at pH 7.0 **(D)**, pH 8.0 **(E)** and pH 9.0 **(F)**. The samples contained 5.64 μmol of enzyme and the spectra were recorded using a Jasco J-810 spectropolarimeter with a 10 mm path length. Similarly, the thermal denaturation of the enzyme was measured at 222 nm as a function of the temperature at pH 7.0 **(G)**, pH 8.0 **(H)** and pH 9.0 **(I)**. The melting temperature (T_*m*_) is shown for each spectrum.

After Est16 purification, we performed a preliminary analysis of its activity against tributyrin in Petri dish assays at different pHs. The results clearly showed that Est16 cleaves tributyrin more efficiently in basic pH (8.0 and 9.0) relative to neutral pH (Fig 5A–5C). On the other hand, the loss of activity was not due to differences in the secondary structure of Est16, which reveals an alpha-beta structure, as is expected for esterases/lipases enzymes [37] (Fig 5D–5F). This result is in agreement with the structural model and with the CD analysis that revealed negative peaks of 210 nm (shift from 208 nm) and 222 nm, characteristics of alpha-beta secondary structure content. In accordance with the higher activity at pH 9.0, Est16 showed two steps for complete unfolding (40°C and 95°C, respectively), revealing a thermally stable structure (Fig 5G–5I).

### Substrate specificity and kinetic parameters of Est16

**Fig 6:**
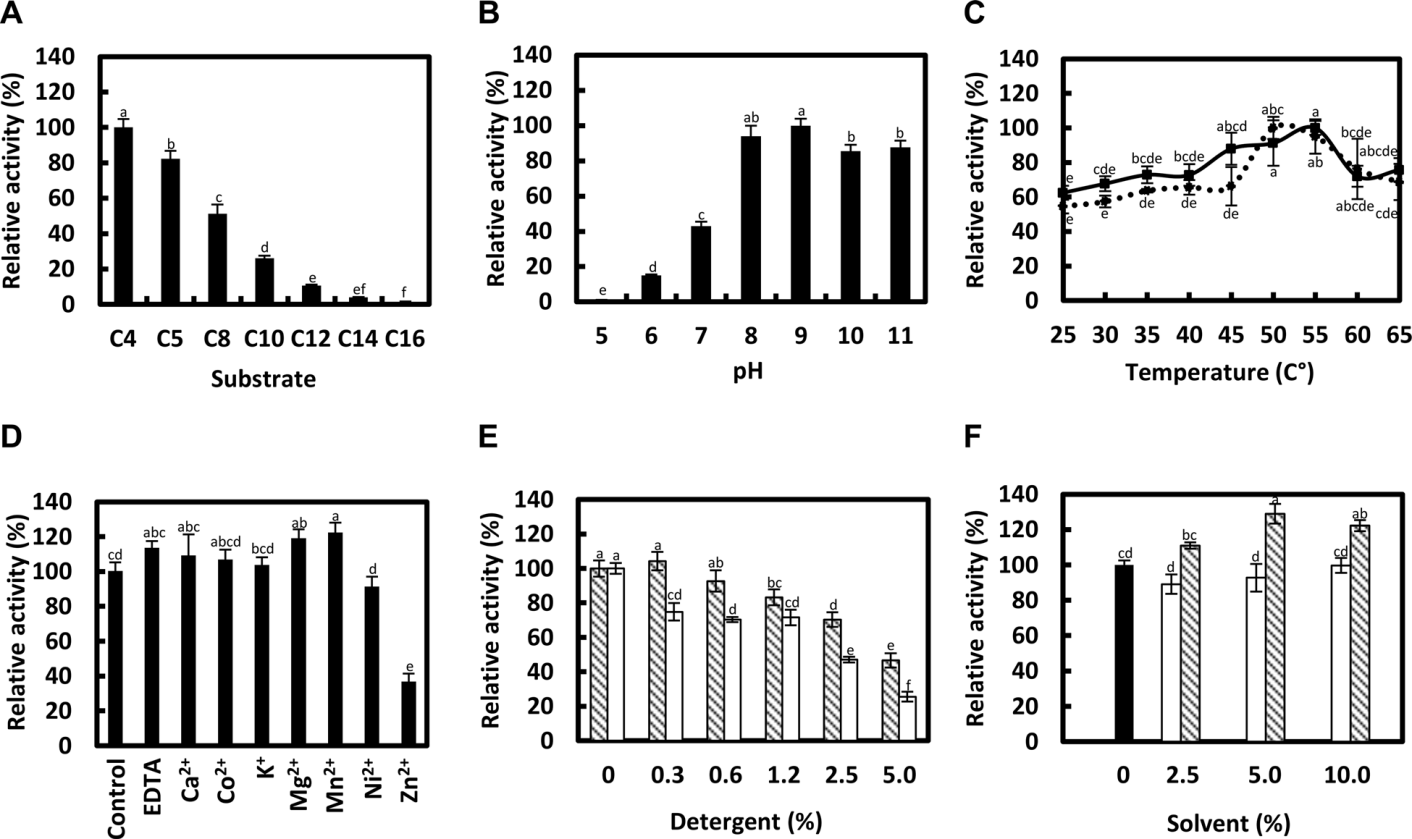
Kinetics analyses of Est16. **(A)** Substrate specificity against *p*-NP esters of various lengths (C_4_ to C_16_); **(B)** The effect of pH on Est16 activity against *p*-NP butyrate; **(C)** The effect of temperature on Est16 activity against *p*-NP valerate (■) and octanoate (●); **(D)** Est16 activity against *p*-NP butyrate was evaluated in the presence of Ca^2+^, Co^2+^, K^+^, Mg^2+^, Mn^2+^, Ni^2+^, Zn^2+^ and EDTA; **(E)** The influence of detergents and **(F)** organic solvents on the enzyme catalytic activity torward *p*-NP butyrate. In E, the striped bar is triton X-100 and the white bar is tween 20. In F, the black bar is the control, the white bar is DMF and the striped bar is DMSO. In the graphics (A-F), the small letters on the top indicates the significant difference between each condition performed in the experiment, according to ANOVA and Tukey’s test at 5% probablility.

The substrate specificity was assessed using *p*-NP esters with different acyl chain lengths (C_4_ to C_12_). Est16 was able to hydrolyze substrates with acyl chains up to 14 carbons and had high catalytic efficiency toward *p*-NP esters with acyl chain lengths of four and five carbons (Fig 6A). A sharp decrease in the activity was observed for acyl chain lengths greater than 10 carbons, although it was observed a relative activity of 10.6% toward *p*-NP dodecanoate, which did not show significant difference with *p*-NP myristate. *p*-NP butyrate and valerate were substrates toward which Est16 had higher catalytic efficiencies (K_cat_.K_*m*_^-1^), approximately 60 and 40 min^-1^.μM^-1^, respectively (Table 1). The catalytic efficiency toward butyrate was 4.5, 12.0, and 39.9 times higher than those of octanoate, decanoate and dodecanoate, respectively. Concerning valerate, the catalytic efficiency was 2.9, 7.9 and 26.6 times higher than those of octanoate, decanoate and dodecanoate, respectively. The preference of Est16 for short chain substrates (C < 10) justified its designation as an esterase.

**Table 1:** Kinetic parameters and maximum enzyme rate (V_max_) of Est16 activity against *p*-nitrophenyl substrates.

Substrate	K_*m*_	V_max_	K_cat_	K_cat_·K_*m*_^-1^
	(μM)	(μM·min^-1^)	(min^-1^)	(min^-1^·μM^-1^)
*p*-NP butyrate	225.1 ± 31.8	206.1 ± 9.1	13211.5 ± 583.3	59.9 ± 8.5
*p*-NP valerate	320.4 ± 32.5	197.3 ± 6.8	12647.4 ± 435.9	39.9 ± 4.0
*p*-NP octanoate	628.6 ± 101.4	127.8 ± 8.6	8192.3 ± 551.3	13.4 ± 2.2
*p*-NP decanoate	1066.0 ± 116.9	82.7 ± 4.5	5301.3 ± 288.5	5.0 ± 0.6
*p*-NP dodecanoate	969.5 ± 42.5	22.9 ± 0.5	1467.9 ± 32.1	1.5 ± 0.1

The effect of pH on the enzymatic activity of Est16 was evaluated with *p*NP-butyrate as a substrate. Est16 was found to be an alkaline esterase that is stable in a broad pH range of 7 to 11, but its maximal activity was at pH 8–9, which no significant difference between them (Fig 6B). Interestingly, Est16 showed activity over the entire temperature range assayed (25 to 65°C) but the optimal temperature for Est16 activity toward *p*NP-valerate and octanoate was 55°C and 50°C, respectively (Fig 6C). This behavior is in agreement with the biophysical studies (Fig 5) and reveals an interesting applicability of this enzyme for biotechnological approaches. The effects of metal ions on the enzyme activity were evaluated by measuring the residual esterase activity in the presence of 0.5 mM of Ca^2+^, Co^2+^, K^+^, Mg^2+^, Mn^2+^, Ni^2+^ and Zn^2+^. The enzyme activity did not show any change in the presence of metals except that Zn^2+^ was observed to induce a 64% reduction in activity relative to the control, being significantly different (Fig 6D). Similarly, we tested the effects of detergents and solvents on the activity of Est16. The reaction rate was measured in the presence of triton X-100, tween 20, DMF or DMSO. Est16 tolerated up to 0.6% (v/v) triton X-100 showing no significant difference with the control (Fig 6E). Additionally, Est16 showed 46% and 25% of relative activity in the presence of 5% (v/v) of both detergents, respectivelly. Interestingly, the enzymatic catalysis was increased in the presence of the highly polar, water-miscible solvent DMSO and was not affected by up to 10% (v/v) DMF, being not significantly different with the control (Fig 6F).

## Conclusions

In this work, a function-based screening of a metagenomic library obtained from oil-contaminated soil was used to search for lipolytic activity. The isolated and characterized enzyme Est16 showed conservation of the sequence and structural features of members from family V of bacterial lipolytic enzymes as well as the patented Anti-Kazlauskas lipase, which shows biotechnological applications in the pharmacological industry. Using structural comparison, spectroscopic and kinetic analyses, we demonstrated that this enzyme has a potential biotechnological application because it shows significant differences in its substrate-binding pocket, it is stable at high temperatures and in the presence of organic solvents, and it also shows a large range of substrates.

## Supporting Information

S1 Fig Phylogenetic tree of Est16 with members representing the eight families of bacterial lipolytic enzymes. **(A)** A phylogenetic tree of 34 sequences from NCBI and the Est16 protein was generated using the Maximum Likehood method (MEGA6). The numbers at the nodes indicate the bootstrap percentages from 1,000 replicates. The estimated value of the shape parameter for the discrete Gamma Distribution is 5.9377. Substitution pattern and rates were estimated under the WAG model (+Gamma +Freq) with 5 categories. **(B)** The amino acid sequence alignment of Est16 with a member of family V [*Sulfolobus acidocaldarius* (AAC67392.1)]. The residues involved in the substrate-pocket and family classification are shown in bold and the catalytic triad residues are denoted with asterisks (*).
(TIF)

S1 Table List of esterases/lipases used for building the phylogenetic tree. The sequences of members representing the eight families of bacterial lipolytic enzymes were obtained from NCBI (http://www.ncbi.nlm.nih.gov) and submitted to the amino acid sequence alignment using ClustalW [29]. The phylogenetic tree was built using MEGA6 [31].
(PDF)

S2 Table List of esterases/lipases used for building the phylogenetic tree of Est16 and the patented enzymes. The patented enzymes were searched for in the literature and the Questel website using the Orbit database. After the patented enzymes were chosen, their sequences were obtained at GenomeQuest website (http://www.genomequest.com/). When available, a PDB code for the solved three-dimensional structures of the enzymes is shown.
(PDF)
