# Supplementary figures and images for: Est16, a New Esterase Isolated from a Metagenomic Library of a Microbial Consortium Specializing in Diesel Oil Degradation
Source: PLoS One. 2015 Jul 27;10(7):e0133723. doi: 10.1371/journal.pone.0133723 (PMC4516351; doi:10.1371/journal.pone.0133723)

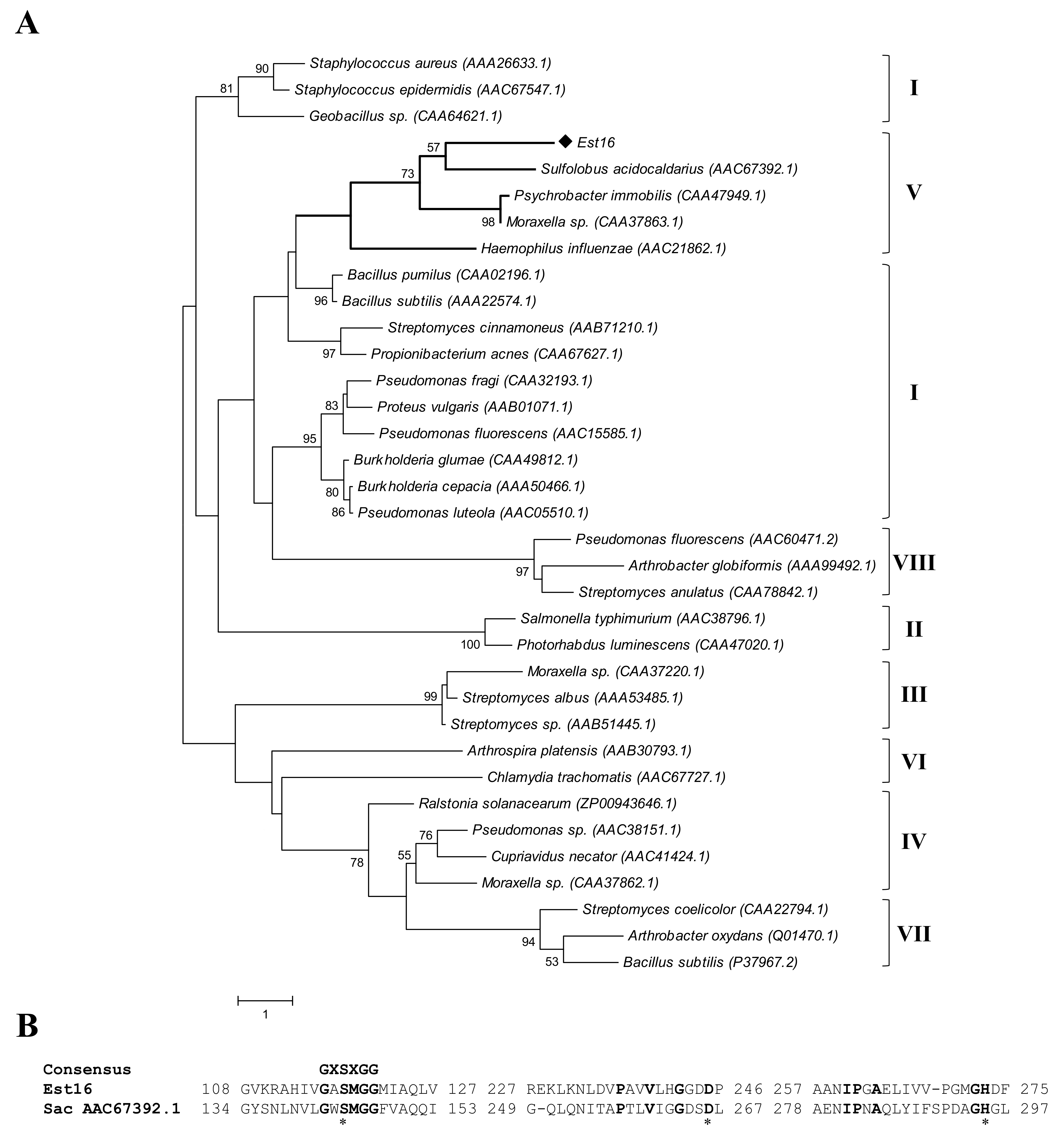

Supplement: S1 Fig — (A) A phylogenetic tree of 34 sequences from NCBI and the Est16 protein was generated using the Maximum Likehood method (MEGA6). The numbers at the nodes indicate the bootstrap percentages from 1,000 replicates. The estimated value of the shape parameter for the discrete Gamma Distribution is 5.9377. Substitution pattern and rates were estimated under the WAG model (+Gamma +Freq) with 5 categories. (B) The amino acid sequence alignment of Est16 with a member of family V [Sulfolobus acidocaldarius (AAC67392.1)]. The residues involved in the substrate-pocket and family classification are shown in bold and the catalytic triad residues are denoted with asterisks (*). (TIF) [file pone.0133723.s001.tif]
