# Supplementary material for: Est16, a New Esterase Isolated from a Metagenomic Library of a Microbial Consortium Specializing in Diesel Oil Degradation
Source: PLoS One. 2015 Jul 27;10(7):e0133723. doi: 10.1371/journal.pone.0133723 (PMC4516351; doi:10.1371/journal.pone.0133723)
Supplement: S1 Table — The sequences of members representing the eight families of bacterial lipolytic enzymes were obtained from NCBI (http://www.ncbi.nlm.nih.gov) and submitted to the amino acid sequence alignment using ClustalW [29]. The phylogenetic tree was built using MEGA6 [31]. (PDF) [file pone.0133723.s002.pdf]

Table S1.

**Access number**	**Microorganism**	**Protein function**	**Blast X PDB Query cover/identity (%)**
AAC05510.1	*Pseudomonas luteola*	Triacylglycerol lipase precursor	1OIL 88/93
AAA50466.1	*Burkholderia cepacia*	Lipase	1OIL 87/94
CAA49812.1	*Burkholderia glumae*	Lipase	2ES4 89/100
AAC15585.1	*Pseudomonas fluorescens*	Lipase	3W9U 98/45
AAB01071.1	*Proteus vulgaris*	Alkaline lipase	3W9U 99/80
CAA32193.1	*Pseudomonas fragi*	Unnamed protein product	3W9U 95/47
CAA64621.1	*Geobacillus thermocatenulatus*	Triacylglycerol lipase	2W22 93/99
AAC67547.1	*Staphylococcus epidermidis*	Lipase precursor	2HIH 64/48
AAA26633.1	*Staphylococcus aureus*	Lipase precursor	2HIH 59/52
AAB71210.1	*Streptomyces cinnamoneus*	Lipase LipA	1YS1 29/37
CAA67627.1	*Propionibacterium acnes*	Triacylglycerol lipase	1T4M 67/26
CAA02196.1	*Bacillus pumilus*	Lipase	1I6W 100/80
AAA22574.1	*Bacillus subtilis*	Lipase	1I6W 85/99
AAC38796.1	*Salmonella typhimurium*	Outer membrane esterase	3KVN 75/28
CAA47020.1	*Photorhabdus luminescens*	Triacylglycerol lipase	3KVN 80/26
CAA37220.1	*Moraxella* sp.	Unnamed protein product	3VIS 69/46

AAA53485.1	*Streptomyces albus*	Lipase precursor	1JFR 85/83
AAB51445.1	*Streptomyces* sp.	Triacylglycerol acylhydrolase	1JFR 84/100
AAC38151.1	*Pseudomonas* sp.	Lipase	1QZ3 99/45
AAC41424.1	*Cupriavidus necator*	Lipase-like enzyme	1QZ3 86/37
CAA37862.1	*Moraxella* sp.	Triacylglycerol lipase	1JJI 76/32
ZP00943646.1	*Ralstonia solanacearum*	Esterase	3V9A 72/46
AAC67392.1	*Sulfolobus acidocaldarius*	Lipolytic enzyme	3E3A 83/27
CAA47949.1	*Psychrobacter immobilis*	Triacylglycerol lipase	4OPM 87/34
CAA37863.1	*Moraxella* sp.	Triacylglycerol lipase	4OPM 88/34
AAC21862.1	*Haemophilus influenzae*	Esterase/lipase	3BF7 88/44
AAB30793.1	*Arthrospira platensis*	Serine esterase	4FHZ 97/32
AAC67727.1	*Chlamydia trachomatis*	Predicted lysophospholipase esterase	4F21 84/24
CAA22794.1	*Streptomyces coelicolor*	Putative carboxylesterase	2OGT 95/38
Q01470.1	*Arthrobacter oxydans*	Phenmedipham hydrolase	2OGT 95/36
P37967.2	*Bacillus subtilis*	Para-nitrobenzyl esterase	1QE3 100/98
AAC60471.2	*Pseudomonas fluorescens*	Esterase III	3ZYT 92/28
CAA78842.1	*Streptomyces anulatus*	Esterase A	3ZYT 89/34
AAA99492.1	*Arthrobacter globiformis*	Carboxylic ester hydrolase	3ZYT 99/81
