# Supplementary material for: Est16, a New Esterase Isolated from a Metagenomic Library of a Microbial Consortium Specializing in Diesel Oil Degradation
Source: PLoS One. 2015 Jul 27;10(7):e0133723. doi: 10.1371/journal.pone.0133723 (PMC4516351; doi:10.1371/journal.pone.0133723)
Supplement: S2 Table — The patented enzymes were searched for in the literature and the Questel website using the Orbit database. After the patented enzymes were chosen, their sequences were obtained at GenomeQuest website (http://www.genomequest.com/). When available, a PDB code for the solved three-dimensional structures of the enzymes is shown. (PDF) [file pone.0133723.s003.pdf]

**Table S2.**

| **Reference code** | **Microorganism** | **Protein function** | **Protein identification/ Patent number** | **Blast X PDB Query cover/ID (%)** |
|---|---|---|---|---|
| Unc_US20050153404_pharma | Unculturable strain | Lipase - preparing of enantiomeric enriched esters and alcohols | US20050153404 | 1Q0R 89/28 |
| Pfl_P22862_1VA4 | *Pseudomonas fluorescens* | Esterase - increasing the already-existing perhydrolase activity | US7384787 | 1VA4 99/100 |
| Pal_WO9530744_detergent | *Pseudomonas alcaligenes* | Lipase - improved resistance to surfactants | WO9530744 | 1EX9 90/81 |
| Sgr_WO2011150157_detergent | *Streptomyces griseus* | Lipase - highly suitable as detergent | WO2011150157 | 3D2C 38/39 |
| Psa_WO2010134035_food | *Pelomonas saccharophila* | Lipase - combined enzymes, preparing bread and baking | WO2010134035 | 1GCY 97/94 |
| Sri_WO2010065455_detergent | *Streptomyces* | Lipase - removal | WO2010065455 | 4HYQ |

| | | | | |
|---|---|---|---|---|
| | *grimosus* | of oily stains from fabric | | 85/65 |
| Ssp_WO2006008653_food | *Streptomyces sp.* | Lipase - hydrolysis of glycolipids and phospholipids | WO2006008653 | - |
| Lre_US20100216212_pharma | *Lactobacillus reuteri* | Lipase - anti-obesity activity | US20100216212 | - |
| Tla_WO2006136159_pharma | *Thermomyces lanuginosus* | Lipase - treatment of human disorders | WO2006136159 | 2YIJ 32/27 |
| Mgr_WO2007096201_food | *Magnaporthe grisae* | Lipase - baking, degumming, food emulsifiers | WO2007096201 | 2YIJ 47/24 |
| Fox_US20030180418_food | *Fusarium oxysporum* | Lipase - doughs or baked products | US20030180418 | 2YIJ 44/25 |
| Unk_WO2009106575_food | Unknown strain | Lipase - dairy or baking industry | WO2009106675 | 2YIJ 45/25 |
| Rsp_WO2007128496_chemicals | *Rhodococcus sp.* | Esterase - production of fine chemicals | WO2007128496 | - |
| Bsp_WO2008032007_biofuel | *Bacillus sp* | Lipase - biodiesel production | WO2008032007 | - |
| Dgr_WO2004064537_food | *Digitalis grandiflora* | Lipase - food emulsifiers | WO2004064537 | 4LIX 30/28 |
| Unk_WO2007128441_chemicals | Unknown | Esterases - | WO2007128441 | - |

| | | | | |
|---|---|---|---|---|
| | | production of fine chemicals | | |
| Mth_WO2012078741_industrial | *Myceliophthora thermophila* | Esterases - industrial food, bioremediation, biorefining, detergents | WO2012078741 | 24/30 |
| Csp_CN101957838_bioremediation | *Culex sp* | Esterases - bioremediation of organic phosphorus | CN101957838 | 4FNM 98/38 |
| Lcu_US5843758_bioremediation | *Lucilia cuprina* | Esterases - bioremediation of organic phosphate and carbamate | US5843758 | 4FG5 100/99 |
